# Overview of Control Programs for Twenty-Four Infectious Cattle Diseases in Italy

**DOI:** 10.3389/fvets.2021.665607

**Published:** 2021-04-26

**Authors:** Marco Tamba, Ivana Pallante, Stefano Petrini, Francesco Feliziani, Carmen Iscaro, Norma Arrigoni, Daria Di Sabatino, Antonio Barberio, Veronica Cibin, Annalisa Santi, Marco Ianniello, Luigi Ruocco, Nicola Pozzato

**Affiliations:** ^1^Istituto Zooprofilattico Sperimentale della Lombardia e dell'Emilia Romagna, Brescia, Italy; ^2^Istituto Zooprofilattico Sperimentale delle Venezie, Legnaro, Italy; ^3^Istituto Zooprofilattico Sperimentale dell'Umbria e delle Marche, Perugia, Italy; ^4^Istituto Zooprofilattico Sperimentale dell'Abruzzo e del Molise, Teramo, Italy; ^5^Ministry of Health, General Directorate of Animal Health and Veterinary Medicinal Products, Rome, Italy

**Keywords:** cattle, control programs, infectious diseases, Italy, SOUND-control project

## Abstract

The cattle industry is a major driving force for the Italian agricultural sector totalling about 5. 6 million heads for dairy and meat production together. It is particularly developed in the northern part of the country, where 70% of the whole Italian cattle population is reared. The cattle industry development in the rest of the country is hampered by the hard orography of the territories and a variety of socioeconomic features leading to the persistence of the traditional rural farming systems. The differences in the farming systems (industrial vs. traditional) also affect the health status of the farms. Whereas, Enzootic Bovine Leukosis (EBL) is almost eradicated across the whole country, in Southern Italy where Bovine Tuberculosis and Brucellosis are still present and Bluetongue is endemic due to the presence of the competent vector (*Culicoides imicola*), less investments are aimed at controlling diseases with economic impact or at improving farm biosecurity. On the other hand, with the eradication of these diseases in most part of the country, the need has emerged for reducing the economic burden of non-regulated endemic disease and control programs (CPs) for specific diseases have been implemented at regional level, based on the needs of each territory (for instance common grazing or trading with neighboring countries). This explains the coexistence of different types of programs in force throughout the country. Nowadays in Italy, among cattle diseases with little or no EU regulations only three are regulated by a national CP: Enzootic Bovine Leukosis, Bluetongue and Paratuberculosis, while Bovine Genital Campylobacteriosis and Trichomonosis are nationwide controlled only in breeding bulls. For some of the remaining diseases (Infectious Bovine Rhinotracheitis, Bovine Viral Diarrhea, *Streptococcus agalactiae*) specific CPs have been implemented by the regional Authorities, but for most of them a CP does not exist at all. However, there is a growing awareness among farmers and public health authorities that animal diseases have a major impact not only on the farm profitability but also on animal welfare and on the use of antibiotics in livestock. It is probable that in the near future other CPs will be implemented.

## Introduction

The European Union (EU) animal health policy covers all animals in the EU kept for food, farming, sport, companionship, entertainment and in zoos. It protects human and animal health and welfare as well as food safety as it is working toward high animal health status of livestock, poultry and fish by controlling animal disease outbreaks and by surveillance and eradication programmes. It ensures smooth and safe internal EU market of live animals and products of animal origin (including animal by-products) (https://ec.europa.eu/food/animals/health_en).

Recently the EU Animal Health Policy has been revised and a new Animal Health Law (AHL) was published in March 2016 (Regulation 2016/429/EU). The AHL enters into force on April 2021 and is based on the EU Animal Health Strategy “Prevention is better than cure.” However, only a list of priority diseases is included in the AHL, excluding several diseases with a significant impact on cattle farms profitability. Besides EU regulations, there is a plethora of national and regional requirements as well as private initiatives which vary among countries. Hence, the regulatory landscape in the EU includes a mixture of animal diseases control activities managed by the public sector, private sector or both (1).

The implementation of disease control programs (CPs) provides benefits for animals, farmers, the industry and the consumers, because CPs increase animal health and welfare and decrease antibiotic use. Control programs reduce direct disease losses (e.g., by decreasing the number of diseased animals and increasing production performance) and indirect disease losses (e.g., consequences of trade constraints) (2).

In this paper we report the current Italian situation on 24 cattle diseases selected in the framework of the SOUND-control project (https://sound-control.eu/) for which specific national or regional surveillance programs have been implemented in other countries throughout Europe (2, 3). All these diseases have little or no EU regulations, because not listed in the AHL as diseases to be eradicated from the whole EU territory (2).

This report does not include data on water buffaloes (*Bubalus bubalis*), because in Italy this species is not considered as cattle regarding CPs.

## The Cattle Rearing System in Italy

More than half of the Italian livestock holdings is represented by cattle farms, with 140,105 active holdings rearing over 5.6 million animals were registered in the National Livestock Register at 30th June 2020 (4).

Cattle farms are not homogeneously distributed throughout the Italian territory, which is divided into 21 administrative units (19 regions and two Autonomous Provinces), grouped in four geographical areas (North, Central, South and islands) (5).

Almost half of the cattle farms (45.8%; 64,174/140,105) is located in the nine northern regions, particularly in the Po Valley, counting for 70.0% of the whole Italian cattle population. Analyzing data in detail (Table 1), it emerges that the highest number of cattle farms (11.1%, 15,505/140,105), is located in Lombardy region, where 26.7% (1,498,742/5,613,386) of the whole Italian cattle population (4) is farmed.

**Table 1:** Cattle distribution in Italy at 30th June 2020.

**Geographical area**	**Regions**	**N° herds**	**N° heads**	**% Herds**	**% Heads**	**Average herd size**
North	Aosta Valley	2,084	32,384	1.5	0.6	15.5
	Piedmont	11,987	814,248	8.6	14.5	67.9
	Liguria	1,079	12,562	0.8	0.2	11.6
	Lombardy	15,505	1,498,742	11.1	26.7	96.7
	AP Bolzano	8,172	124,888	5.8	2.2	15.3
	AP Trento	1,537	44,719	1.1	0.8	29.1
	Veneto	15,033	752,962	10.7	13.4	50.1
	Friuli Venezia Giulia	2,203	74,395	1.6	1.3	33.8
	Emilia-Romagna	6,574	571,955	4.7	10.2	87.0
	*North Subtotal*	*64,174*	*3,926,855*	*45.8*	*70.0*	*61.2*
Central	Tuscany	3,780	88,259	2.7	1.6	23.3
	Umbria	3,229	55,461	2.3	1.0	17.2
	Marche	3,521	46,878	2.5	0.8	13.3
	Latium	11,989	199,753	8.6	3.6	16.7
	*Central Subtotal*	*22,519*	*390,351*	*16.1*	*7.0*	*17.3*
South	Abruzzo	4,090	63,107	2.9	1.1	15.4
	Molise	2,486	38,570	1.8	0.7	15.5
	Campania	10,591	163,338	7.6	2.9	15.4
	Apulia	4,226	176,910	3.0	3.2	41.9
	Basilicata	2,714	100,456	1.9	1.8	37.0
	Calabria	8,777	117,246	6.3	2.1	13.4
	*South Subtotal*	*32,884*	*659,627*	*23.5*	*11.8*	*20.1*
Islands	Sicily	11,187	358,744	8.0	6.4	32.1
	Sardinia	9,341	277,809	6.7	4.9	29.7
	*Islands Subtotal*	*20,528*	*636,553*	*14.7*	*11.3*	*31.0*
	Total Italy	**140,105**	**5,613,386**	**100**	**100**	**40.1**

The remaining 30% of the national cattle livestock is held in the 12 regions of Central and South Italy and in the two main islands (Sicily and Sardinia). Among these regions, Lazio holds the highest number of farms and Sicily rears the highest number of animals, 8.0 and 6.4%, respectively (4).

Lombardy and Emilia-Romagna regions show the highest herd size, with an average number of heads per farm of 96.7 and 87.0, respectively (Table 1).

**Figure 1:**
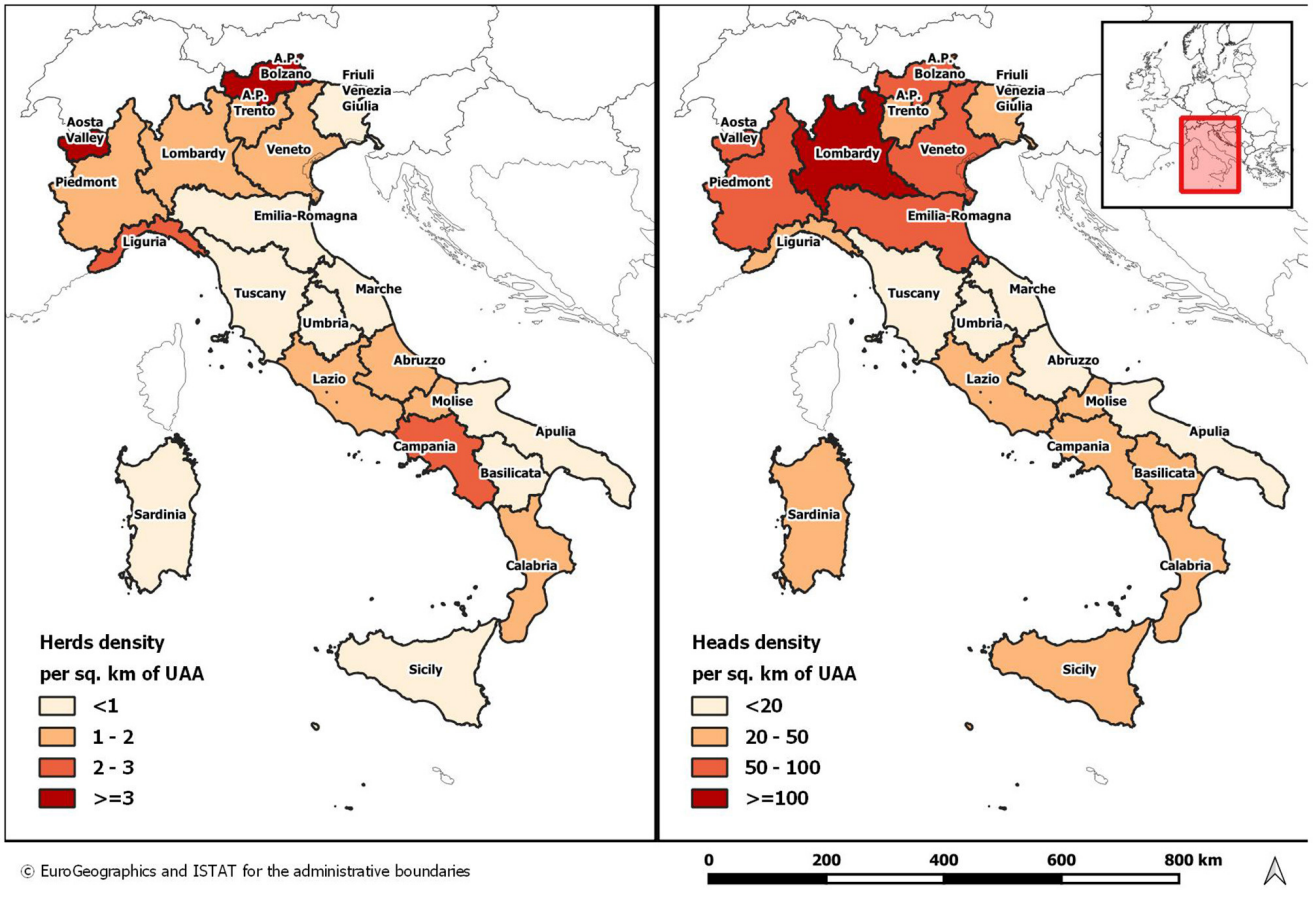
Average density of Italian cattle population per km^2^ of Utilized Agricultural Area (UAA) as of 30th June 2020.

Figure 1 shows that the density of herds and heads per square kilometer of Utilized Agricultural Area (UAA) is not uniform throughout the country. The density of heads is much lower in the regions of central and Southern Italy, including the islands, where extensive grazing farms are prevalent.

The Italian cattle population is composed of: 26,255 dairy farms (18.7%) with a total of 2,626,812 animals (46.8%), 95,478 beef farms (68.2%) with 2,468,849 animals (44.0%) and 18,360 mixed cattle farms (13.1%), rearing 517,709 (9.2%) animals (4).

**Figure 2:**
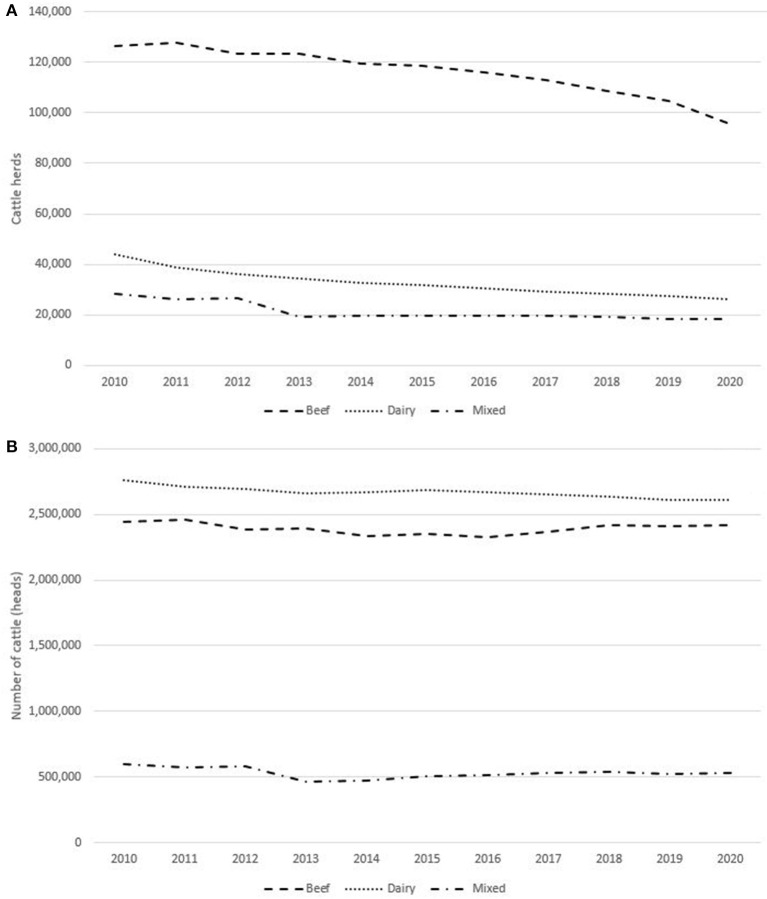
Trends in cattle populations (**A** herds, **B** animals), Italy 2010–2020.

The last 11 years (2010–2020) have shown a decreasing trend in the number of cattle farms (Figure 2A), mainly dairy farms (from 44,109 to 26,255; −40%). The same scenario has also been observed for both beef and mixed cattle farms, even if at a minor extent (−24 and −35%, respectively). Despite the reduction in the number of holdings, the cattle population has not proportionally decreased (Figure 2B). As a consequence in Italy the average herd size has increased in the same period from 29.2 to a 40.1 heads.

Friesian and Brown Swiss are the most frequent breeds kept in dairy farms, with 1,972,165 (75.1%) and 99,157 heads (3.8%), respectively, while in beef farms the most frequent breeds are mixed-breed and Piemontese, with 950,859 (38.5%) and 313,252 (12.7%) heads, respectively.

Three types of farming method are recorded in the National Cattle Database (4): intensive, extensive, and farms doing transhumance (seasonal movement to pasture). Unfortunately, over half of the Italian farms (58.1%; 81,457/140,105) have not a registered farming method because this information is not mandatory. However, among those (49.1%) for which the farming method is registered, 22.2% are intensive farms, 16.1% are extensive farms and only 3.6% are farms doing the transhumance (mostly located in the alpine area).

Different geographical, social and economic characteristics could explain why some farming methods are more common in some areas of the country. The predominance of mountainous territory can justify the necessity of doing transhumance while in regions with abundant pasture, it is common to raise animals in extensive farms, in contrast to those regions where the UAA is limited, and only intensive farming is feasible.

### Cattle Trading

Italy is a strong importer of live cattle for fattening and beef meat (fresh, chilled or frozen, intended for consumption or subsequent industrial processing). In 2013 the import value covered, approximately 42 and 58% of the total national demand of live cattle for fattening and beef, respectively.

Trading trends from other countries are mainly characterized by: (i) a decrease in the import of live animals for both fattening and slaughter, (ii) an increase in the import of fresh meat, (iii) a reduction in import of frozen and preserved meat from all countries (6).

**Figure 3:**
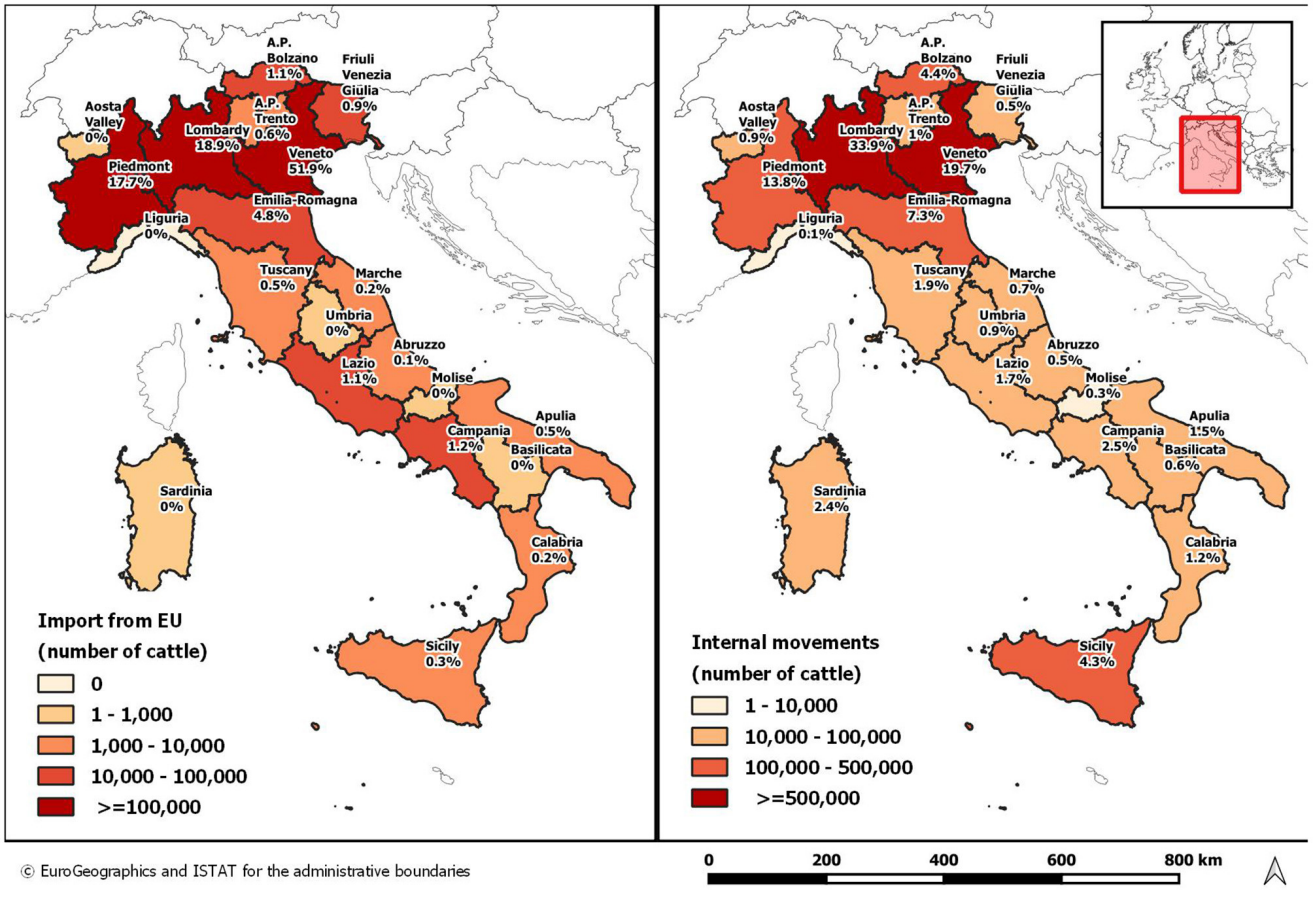
Distribution of import and internal cattle movements, by region of destination, Italy 2019.

In 2019, 1,147,307 live cattle have been imported in Italian holdings, mainly located in Veneto (51.9%; 595,545/1,147.307), followed by Lombardy (18.9%), Piedmont (17.7%) and Emilia Romagna (4.8%) (Figure 3). The majority of the imported cattle came from France (943,867 heads; 82.3%). The remaining animals were introduced from Austria, Ireland, Germany, Poland, Lithuania, Romania and Spain.

Moreover, cattle (mainly beef calves) moved between Italian farms are primarily destined to Lombardy and Veneto regions (Figure 3).

Relative to movements for pasture, 430,819 animals were moved in 2019, mainly in the alpine regions. These figures have been constant, on a yearly basis, in the last 10 years.

Export of cattle is almost insignificant: in 2019, only 17,077 heads were exported, a yearly rate constant in the last decade. Veneto and Lombardy are the most exporting regions with 41.9 and 38.9% of total exported cattle, respectively. Almost half of the cattle has been exported to Romania (30.9%) and Spain (15.5%) (4).

### Welfare and Biosecurity on Cattle Farms

Welfare and biosecurity assessment are performed regularly by official veterinary services in beef and dairy cattle according to the national plan for welfare in farm animals. The assessment is performed by mean of a specific checklist that includes: animal management, housing and feeding systems, and animal based measures (ABMs). ABMs evaluation is performed according to the methodology developed by the National Reference Center for Animal Welfare (CRENBA), and include several scores relative to animal conditions as: cleanliness, body condition score, lameness score and integumentary system lesions (7). The minimum number of herds to be controlled are defined by the “national plan for animal welfare assessment,” and in the last years the target was fixed at 10% of the entire Italian farms. Recently the Ministry of Health has introduced a ranking system for cattle herds, based on voluntary adhesion by the farmers, called “Classyfarm.” The farmers that apply to “Classyfarm” should perform an assessment of animal welfare and biosecurity conditions through the checklist developed by CRENBA filled by veterinary practitioners specifically trained for this activity. For each adhering farm, the data are recorded and analyzed to provide a rank of the animal welfare and biosecurity condition of the farm. Data about drugs consumption are also recorded in the same system. Official veterinary authorities use all these data to perform herd risk assessments and to plan risk-based official controls.

### Diseases Controlled in Breeding Bulls

Specific programs are provided for breeding bulls. Bulls approved for natural breeding shall belong to farms officially free from Bovine Tuberculosis, Brucellosis and Leukosis. Before breeding they have to be negative to the following tests:

- an intradermal tuberculin test for Bovine Tuberculosis;
- a serological test for Bovine Brucellosis;
- a serological test for Enzootic Bovine Leukosis;
- a serological test for Infectious Bovine Rhinotracheitis (whole virus);
- a serological test for Bovine Viral Diarrhea;
- a microscopic test for Trichomonosis.

These checks are repeated every year.

There are only a few bulls (males older than 24 months) in Italian cattle farms. At 30th of June 2020 there were, respectively, 8.142 bulls in dairy holdings (one bull every 3.2 farms) and 51,864 in beef and mixed operations (one bull every 2.2 farms). In Italy it is not customary to exchange bulls between farms, and neither public nor private stations for natural breeding are working.

Artificial insemination is widely applied. In Italy there are 87 semen collection centers (1,178 heads). Within 28 days before entering the semen collection center, bulls, belonging to farms officially free from Bovine Tuberculosis, Brucellosis and Leukosis, are checked for the following diseases, according to Directive 88/407/EEC (8):

- an intradermal tuberculin test for Bovine Tuberculosis, with a negative result;
- a serological test for Bovine Brucellosis, with a negative result;
- a serological test for Enzootic Bovine Leukosis, with a negative result;
- a serological test for Infectious Bovine Rhinotracheitis, with a negative result;
- a virological test for Bovine Viral Diarrhea, with a negative result;
- a serological test for Bovine Viral Diarrhea;
- no signs of skin infectious diseases (Mange, Papillomatosis, Trichophytosis);
- a serological test for Paratuberculosis to the dam of the bull, with a negative result.

Upon arrival at the semen collection center, bulls are kept in quarantine and checked for the following diseases:

- an intradermal tuberculin test for Bovine Tuberculosis, with a negative result;
- a serological test for Bovine Brucellosis, with a negative result;
- a serological test for Enzootic Bovine Leukosis, with a negative result;
- a serological (whole virus) test for Infectious Bovine Rhinotracheitis, with a negative result;
- a virological test for Bovine Viral Diarrhea, with a negative result;
- a serological test for Bovine Viral Diarrhea, applied only to animals resulted seronegative to the check before entering, with no seroconversion detected;
- a virological and a serological test for Bluetongue, with negative results;
- a microscopic test on a sample of preputial washing for Trichomonosis, with negative results;
- a bacteriological test on a sample of preputial specimen for Bovine Genital Campylobacteriosis.

The same program is carried out every year on bulls kept in the artificial insemination centers. Bulls with a positive result are removed from the center.

## The Control System of Cattle Diseases in Italy

The Directorate-General for Animal Health and Veterinary Medicine (DGAHVM) of the Ministry of Health is the Italian Central Competent Authority (CCA) for animal health. DGAHVM is responsible for drawing up national plans, which must then be implemented by the 21 regional authorities of the country. The CCA carries out a systematic verification and monitoring of the financial aspects of the national surveillance (control and/or eradication) programs, currently covering the following cattle diseases: Bovine Brucellosis, Enzootic Bovine Leukosis, Bovine Tuberculosis, Bluetongue, and Bovine Spongiform Encephalopathy.

The Italian Regions are coordinated and administratively controlled by the CCA, even if the CCA does not have the authority to modify regional policies. Thus, Regions may adopt their own programs on animal diseases not regulated at national level, providing only an informational notification to the CCA. Consequently, Italy has a plethora of control programs for cattle diseases put in place to address different regional interests such as: export of local animal products, grazing, trade of live animals with neighboring countries where a national control plan is already mandatory (e.g., Austria, Switzerland, France).

A network of ten public laboratories called Istituti Zooprofilattici Sperimentali (IIZZSS), in which all the National Reference Centers for Animal diseases are set, provides the diagnostic services to the official veterinary services, which are responsible for the implementation of animal diseases control programs (9).

## Cattle Diseases With a Control Program in Italy

The following diseases are covered by a control program at least at regional level. According to the Italian regulation they are divided into two categories: (i) notifiable diseases and (ii) non-notifiable diseases. For notifiable diseases, specific sanitary measures are compulsorily applied in case of an outbreak (10). As a general rule, in Italy only cattle farms with breeders are included in the control programs, whereas farms consisting only of fattening animals (calves and steers) are excluded.

### Enzootic Bovine Leukemia

Enzootic Bovine Leukemia (EBL) is a notifiable disease. Since 1996, Italy has adopted an eradication program in compliance with EU regulations (Council Directive 97/12/EC). The program is based on the “test and removal” strategy; which is the only one applicable as neither a vaccine nor an effective therapy is available (11).

The eradication program is mandatory and fully financed by the Government. Every year in breeding farms, all cattle older than 12 months are tested by serological tests. In the past the reference test was the agar gel immunodiffusion (AGID), but currently an enzyme-linked immunosorbent assay (ELISA) is used, due to its higher sensitivity (12).

Positive animals are considered infected and promptly culled with compensation to the farmer. Moreover, the infected herds are repeatedly controlled to confirm the absence of further cases (10).

A territory achieves the status of officially free from EBL if the herd prevalence decreases below 0.2%. In 2017, the European Commission declared Italy as EBL officially free country (Implementing Decision 2017/1910/EU), despite the presence of some infection clusters located in four regions of Central-Southern Italy (Latium, Apulia, Campania, Sicily). In these clusters, specific additional programs have been applied: all animals older than 6 months are tested and more severe measures are adopted in terms of biosecurity and animal registry (e.g., electronic identification of animals). In these clusters in fact, some factors are delaying the eradication process: free ranging animals, promiscuous breeding of herds, lack in the collaboration of breeders and unrecorded animal movements.

Currently EBL virus complete eradication (0% prevalence) is close to be achieved in South Italy, while only a cluster in the region of Latium is still active (13). In 2020, in Italy 11 EBL positive farms out of 16,960 (0.06%) tested were registered (Table 2).

**Table 2:** Number of Enzootic Bovine Leukosis notified by area. Italy, 2011–2020 (updated to 13/01/2021).

**Year**	**North Italy**	**Central Italy**	**South Italy**	**Islands**	**Italy**
	**Number of outbreaks**	**Number of outbreaks**	**Number of outbreaks**	**Number of outbreaks**	**Number of outbreaks**
2011		6	14	13	33
2012		1	13	17	31
2013		6	11	5	22
2014		6	23	11	40
2015		2	12	3	17
2016		7	10	2	19
2017		4	7		11
2018		5	5		10
2019		5	4		9
2020		10	1		11
Total	0	52	100	51	203

After the EBL official free status achievement in 2017, each region has issued its own surveillance program, which, once approved by the Ministry of Health, has been carried out in the area of competence. The application of surveillance programs on a sampling basis and adapted to the regional situation has resulted in significant economic savings. The Ministry of Health has issued some guidelines to be applied in the 5-years period 2018/2023, in order to standardize the surveillance activities at the national level. All regions must carry out surveillance activities giving evidence of absence of EBL circulation. For this purpose the Ministry of Health has also implemented a dedicated information system collecting, at national level, all the data on EBL control activities (4, 14).

### Bluetongue

BT is a notifiable disease in Italy, and 13,641 outbreaks, out of which 2,287 in cattle farms, have been officially notified in the last 10 years (2011–2020). BT was first detected in 2000, and is currently considered endemic in South Italy and islands, where the highly competent vector *Culicoides imicola* is present. Some BT incursions were registered over time also in North and Central Italy (Table 3). Several Bluetongue virus (BTV) serotypes have been circulating in Italy since the first incursion. In the last 5 years BTV-1, BTV-3 and BTV-4 circulation was confirmed, but only monovalent and/or bivalent vaccines against serotypes 1 and 4 are available at the moment. Considering the available resources and the cost-benefit analyses made by the regional veterinary authorities, the vaccination against the circulating BTV serotypes was carried out in compliance with regional programs. In 2019, vaccination against BTV-4 of all the restocking sheeps was performed only in Sardinia in order to reduce the impact of the mortality due to the disease. The other Italian regions have limited the vaccination of the susceptible animals to those to be moved toward free territories or areas under restriction for different serotypes. The BT Italian surveillance system includes a passive surveillance and a serological program based on sentinel animals. Since 2002, a robust and organized network of sentinel animals has been established in Italy to monitor BTV circulation. The Italian territory has been divided in square grids of 20 × 20 km. In each square, around 58 susceptible animals are selected and used as sentinels. The network was based on more than 30,000 sentinels, checked every month. Since 2019, in response to the new epidemiological situation a new surveillance program was established. The entire Italian country has been divided in square grids of 45 × 45 km and in each cell 59 seronegative animals have been selected and used as sentinels, and quarterly serologically tested. About 9,000 sentinels are periodically tested by c-ELISA. Positive results are confirmed by virus neutralization assay against 10 BTV serotypes (BTV-1, BTV-2, BTV-3, BTV-4, BTV-6, BTV-8, BTV-9, BTV-14, BTV-15, BTV-16). From serologically positive animals, EDTA blood samples are also collected and tested by RT-PCR for the presence of BTV RNA. Virus isolation and typing is also performed in all RT-PCR positive sentinels and animals showing clinical signs.

**Table 3:** Number of Bluetongue outbreaks notified in cattle farms, by area. Italy, 2011–2020 (BT serotyes involved are in brackets). Updated to 13/01/2021.

**Year**	**North Italy**	**Central Italy**	**South Italy**	**Islands**	**Italy**
	**Number of outbreaks**	**Number of outbreaks**	**Number of outbreaks**	**Number of outbreaks**	**Number of outbreaks**
2011			1 (2)	10 (2, 9)	11
2012			6 (1)	10 (1, 2, 9)	16
2013	1 (1)	37 (1)	5 (1, 2, 4)	366 (1, 2, 16)	409
2014	13 (1)	136 (1)	385 (1, 4)	85 (1)	619
2015	1 (1)	24 (1, 4)	105 (1, 4)	37 (1, 4)	167
2016	342 (4)	51 (1, 4)	197 (1, 4)	92 (1, 4)	682
2017	25 (4)	21 (1, 4)	35 (1, 4)	130 (1, 4)	211
2018	4 (1, 4)	3 (1)	13 (1, 4)	34 (1, 3, 4)	54
2019	3 (4)		15 (1, 4)	29 (1, 3, 4)	47
2020	1 (4)	11 (4)	46 (4)	13 (1, 4)	71
Total	390	283	808	806	2,287

Confirmed seroconversions of sentinel animals as well as confirmed clinical cases cause an outbreak notification and the establishment of a restriction zone. Movements of unvaccinated animals are strictly regulated, in restriction zones, to avoid the spreading of the disease.

Moreover, an entomological surveillance system focused on the detection and quantification of *Culicoides* spp. is carried out through fixed black light traps distributed in the whole country and activated on a weekly basis. This surveillance system is used to define seasonal free areas, useful to facilitate animal movements, and to monitor vector dynamics.

### Bovine Paratuberculosis (Johne's Disease)

Bovine Paratuberculosis (JD) is widespread in Italy, where over 50% of bovine dairy herds are infected (15). In order to improve the health status of dairy herds and to protect the dairy export market, the Italian Ministry of Health issued in 2013 the “National guidelines for the control of Bovine Paratuberculosis and for assigning the health ranking of herds.” The guidelines have been adopted by all the Italian regions.

The program has been initially industry-driven and is managed through the collaboration of official veterinary services, bovine practitioners and the network of IIZZSS laboratories. The main components of the program are (16):

- A passive surveillance system with mandatory reporting of clinically affected cows to the official veterinary services. Following the notification, a serological control on all animals older than 36 months is carried out by the official veterinary services free of charge for the farmer.
- The voluntary adoption of herd control programs by the farmers, aimed at gradually reducing within-herd prevalence by adopting biosecurity measures and a standardized testing scheme. The Guidelines provide some tools facilitating the risk assessment of the herd and suggesting the most appropriate measures to be adopted.
- A ranking of bovine herds based on the risk of JD infection in the herd. There are seven JD status levels, the first two are assigned by the official veterinary services on the basis of presence (PTC) or absence (PT0) of confirmed clinical cases of Paratuberculosis. The PTC level identifies those herds that, having had a clinical case in the last 12 months, are not allowed to sell milk for production of export dairy products. The achievement of the further levels (from PT1 to PT5) is obtained upon a specific request of the farmer. The health status of the herd is based on results of a standardized serological testing scheme voluntarily applied every year. PT1 rank corresponds to a seroprevalence <5%; PT2 to a seronegative herd. The higher levels PT3-PT5 are assigned to herds considered free from JD with an increasing level of confidence.

The voluntary certification process is not yet widely applied, more precisely in North Italy at the end of 2019 there were 980 farms certified as JD-free (Table 4).

**Table 4:** Report of the Paratuberculosis status of cattle farms, by area, Italy, 2019.

**JD Status** **of the farm**	**JD status Code**	**North Italy**	**Central Italy**	**South Italy**	**Islands**	**Italy**
		**Number of farms**	**Number of farms**	**Number of farms**	**Number of farms**	**Number of farms**
With JD clinical cases	PTC	25	2	0	9	36
Without JD clinical cases	PT0	28,240	642	571	10,957	40,410
JD low risk (*P* <5%)	PT1	1,291	26	0	49	1,366
JD negative	PT2	3,965	40	4	311	4,320
JD free	PT3	519	3	0	0	522
	PT4	307	4	0	0	311
	PT5	147	0	0	0	147
Not Classified		29,560	21,960	32,493	9,051	93,064
Total		64,054	22,677	33,068	20,377	140,176

### Infectious Bovine Rhinotracheitis

Infectious Bovine Rhinotracheitis (IBR) is not a notifiable disease in Italy, but the disease is widely diffused in the Italian cattle population. Currently, several regional control programs are in place in the country. IBR control programs started in North Italy at regional or provincial levels in the last decade of the past century, to facilitate trading and face restrictions on seasonal movements to the alpine pastures. These territories include four regions, out of which two (Friuli Venezia Giulia and the autonomous province of Trento) have eradication programs approved by the European Commission and are close to IBR eradication. The remaining ones (Valle d'Aosta and the autonomous province of Bolzano) have been recognized as officially IBR-free territories since 2017 (Commission Implementing Decision (EU) 2017/888 amending Dec. 2004/558/EC, Annex II). Since then, other Italian territories developed voluntary control programs. In the region of Piedmont, a voluntary CP started in 2003 partially funded by the regional authority. The CP was updated in 2017 and different sampling schemes are now applied depending on the type of the cattle farm involved:

- In dairy herds, pooled (30–40 cows) milk samples are collected and tested every 5–7 months. Moreover, bulls and other non-producing-milk cattle older than 24 months are serologically checked once a year.
- In beef herds rearing the Piemontese breed, individual blood samples are collected from breeding cattle older than 12 months once a year.
- In the other types of farm, individual blood samples are collected from breeding cattle older than 24 months once a year.

All collected samples are tested by ELISA, and only the use of gE deleted marker vaccines are allowed. At the end of 2018 about 80% (7,219/8,970) of farms joined the regional program, and an IBR prevalence of 15.3 and 3.8% were registered at herd and animal level, respectively, while IBR free herds were the 76.3% of the adherent farms (17).

A similar voluntary program is in place in the region of Lombardy. The program started in 2005 and was updated in 2016. There are some differences with the Piedmont program: pooled milk samples are composed by a maximum of five cows, and the minimum age for serological test is 9 months.

Moreover, in all the territory of the Lombardy region a pre-moving IBR test is compulsory and seropositive animals cannot be moved to another farm. A surveillance program is yearly carried out on non-adherent farms through bulk milk test or individual serological testing of a sample of animals (expected prevalence 5%, confidence intervals 95%). At the end of 2019 in Lombardy a IBR prevalence of 18.3% (1,322/7,218) and 6.0% were registered at herd and animal level, respectively (source: IZSLER).

Furthermore, in 2015 and 2016, the Italian Ministry of Agriculture, Food and Forestry (MIPAAF) and the Italian Ministry of Health approved two surveillance voluntary programs for controlling IBR at national level in farms registered in the two National Herd Books of some indigenous beef cattle breeds: (i) Marchigiana, Romagnola, Podolica, Chianina, and Maremmana breeds; (ii) Piemontese breed. In 2019, the two National Herd Books contained 9,407 herds, representing 6.5% of all cattle herds.

Farmers voluntarily joining the program must test all their breeding cattle aged more than 12 months. Individual blood samples are collected by the official veterinary services, submitted to an IIZZSS laboratory, and tested for the presence of antibodies to glycoprotein E (gE) of BoHV-1 or antibodies to the whole virus of BoHV-1 using commercial enzyme-linked immunosorbent assay kits. There is no IBR confirmation test in the plans. The farmers have to pay for the sampling and testing; but a monetary reward is provided if they achieve the annual target seroprevalence (18). These programs do not include aggressive measures such as culling of positive animals or vaccination. In 2019 in the framework of these programs, 2,972 herds and 132,995 animals were tested; herd seroprevalence was 30.6%, while animal seroprevalence was 8.1%. Herd prevalences were higher in South (64.3%) and Central (43.4%) than in North Italy (25.3%).

The annual results of the above voluntary programs are available at the following link: http://www.izsum.it/IZSUM/Common/pages01/wfEventLink.aspx?IDMAP=631.

### Bovine Viral Diarrhea

Like IBR, Bovine Viral Diarrhea (BVD) is not a notifiable disease in Italy, but it is widely diffused in the Italian cattle population and controlled by farmers through vaccination.

Currently in Italy only a few provinces/regions of the North of Italy have implemented local BVD control or eradication programs funded by competent authorities. They are voluntary or compulsory, depending on regional regulations. In most cases, they have been implemented for trading with neighboring BVD-free countries. These programs are focused on the detection and removal of persistently infected (PI) animals.

Control programs for BVD were first carried out at territorial level by Bolzano and Trento Provinces (north-east of country) in the years of 1999–2000, in order to obtain disease eradication. In these compulsory programs, in fact, vaccination is not allowed. For testing purpose, an antigen detection test (ELISA or PCR) is performed by using ear-notch samples at birth (or serum samples in alternative) in all breeding herds, and positive animals are slaughtered within 3 weeks. In order to move among provincial farms, as well as to participate in cattle shows, a virological test must be performed for all animals, with negative results.

Since 2002 another compulsory program has been implemented in Friuli Venezia Giulia Region (north east of Italy). It provides carrying out an antigen detection test (ELISA) in ear-notch samples at birth (or serum samples in alternative) in all breeding herds and culling of positive cattle as soon as possible. Only breeding cattle that tested negative for antigen detection can be moved from the farm. In infected herds, vaccination is allowed in the 2 years following the removal of the latest PI animal.

Over time, other regions have implemented monitoring programs aimed at estimating the BVD prevalence. Unfortunately, no data have been published. Generally, all epidemiological data about local BVD control programs are collected by Regional authorities, but neither the CCA nor the National Reference Centre are aware of the current status of the obtained results.

### Streptococcus agalactiae

The disease caused by *S. agalactiae* (STAG), called in Italy Contagious Catarrhal Mastitis, is a notifiable disease, but despite its high occurrence in the cattle population, only 28 outbreaks have been officially notified in the last 10 years (2011–2020) (Table 5). In infected herds, cows with clinical mastitis shall be isolated and treated until full recovery. During this period their milk is not allowed for selling on market and for feeding calves (10).

**Table 5:** Number of outbreaks of clinical mastitis caused by *Streptococcus agalactiae* notified in Italy, 2011–2020 (updated to 13/01/2021).

**Year**	**North Italy**	**Central Italy**	**South Italy**	**Islands**	**Italy**
	**Number of outbreaks**	**Number of outbreaks**	**Number of outbreaks**	**Number of outbreaks**	**Number of outbreaks**
2011–2015	14			3	17
2016–2020	5	4	1	1	11
Total	19	4	1	4	28

Although STAG infection has a high economic impact for dairy farms, only two regions in the north of Italy have currently implemented a CP. In the region of Lombardy, a control program was started in 2012, and was updated in 2015 with the aim to reduce the STAG herd prevalence below 8%. All dairy herds are controlled by the official veterinary services. Bacteriological tests are yearly performed on bulk milk and herds with at least five consecutive negative tests are classified as free. In positive farms a voluntary herd eradication program can be applied by the farmer at his own expenses. At the end of 2018, 368 dairy farms (7.3%), out of 5,049 tested, resulted infected (19).

A similar program has been implemented for a 2-year period (2019–2020) by the region of Emilia-Romagna with the following goals:

- estimating the prevalence of STAG in dairy herds of the region;
- reducing of at least 10% the prevalence of STAG infected farms;
- reducing the antimicrobial use in dairy farms.

The program is compulsorily performed by the official veterinary services and funded by the regional authority. Bulk milk of all dairy farms is tested every 6 months for STAG presence. In positive farms a voluntary herd eradication program can be applied by the farmer at his own expenses. However, in infected farms producing milk for direct human consumption, it is mandatory to stop the sale of raw milk until the successful treatment of all infected cows.

Only bulk milk negative farms which have carried out an individual bacteriological check of all the cows with negative results could be certified as free from STAG infection. At the end of 2019 (first year of the program) a STAG prevalence of 8.1% (325/2,848) was scored in the dairy herds of the region of Emilia-Romagna (source: IZSLER).

## Cattle Diseases Without a Control Program in Italy

The following diseases do not have a control program. According to the Italian regulation they are divided into two categories: (i) notifiable diseases and (ii) non-notifiable diseases. For notifiable diseases, specific sanitary measures are compulsorily applied in case of an outbreak (10).

### Anthrax

Anthrax is a notifiable disease in Italy, and 52 outbreaks, out of which 38 in cattle farms, have been officially notified in the last 10 years (2011–2020). The disease is sporadic in regions of Central Italy, islands and south of the country (Table 6). In contrast, in North Italy only a few cases were recorded and genotyping of isolated strains related this occurrence to animal introductions from abroad (20).

**Table 6:** Number of Anthrax outbreaks notified in Italy, 2011–2020 (updated to 13/01/2021).

**Year**	**North Italy**		**Central Italy**		**South Italy and Islands**		**Italy**	
	**Number of Anthrax outbreaks**							
	**All animal species**	**Cattle only**	**All animal species**	**Cattle only**	**All animal species**	**Cattle only**	**All animal species**	**Cattle only**
2011					26	20	26	20
2012			1	1	2	1	3	2
2013							0	0
2014					3	3	3	3
2015					1		1	0
2016			1		6	4	7	4
2017			1	1	1		2	1
2018			1	1	3	2	4	3
2019					3	3	3	3
2020					3	2	3	2
Total	0	0	4	3	48	35	52	38

A vaccine based on attenuated strain Sterne 34F2 is produced by the National Reference Centre for Anthrax (IZSPB, Foggia, Italy) and used for immunization of at risk animals under the Ministry of Health authorization (10, 21).

### Bovine Genital Campylobacteriosis

Bovine Genital Campylobacteriosis (BGC) is not a notifiable disease in Italy. There are no official data about the prevalence of *Campylobacter fetus* subspecies *fetus*.

In Italy, all breeding bulls before entering a reproductive center for semen collection must be kept in quarantine and must test negative to a cultural test for the detection of BGC in samples of preputial material. During the quarantine, bulls younger than 6 months are tested once for BGC, while bulls older than 6 months are tested three times at 1-week intervals. All infected bulls are removed from the semen collection centers. In addition, bulls approved for natural breeding must be yearly tested for the detection of BCG.

Several commercial vaccines are available for BGC, but they are not licensed in Italy and their use in farms must be authorized by the Ministry of Health.

### Trichomonosis

Bovine Trichomonosis is a notifiable disease in Italy, and no outbreaks have been officially notified in cattle in the last 10 years (2011–2020). However, in infected herds natural breeding is stopped and only artificial insemination can be performed. All infected animal must be detected, treated and excluded from natural breeding until the full recovery (10). All bulls approved for public or private breeding must be yearly tested, showing negative results to microscopic and cultural tests for the detection of *Trichomonas foetus*, in samples of preputial material or artificial vaginal liquid lavage (10).

### Salmonella

Clinical Salmonellosis is a notifiable disease in Italy; in case of an outbreak all animal movements are officially blocked until the recovery of all the affected animals (10).

In the last 10 years (2011–2020) 118 outbreaks have been officially notified in cattle, showing an increasing trend starting from 2018 (Table 7); nevertheless it is not possible to exclude that the effectiveness of notifications varies among different regions, thus underreporting is possible.

**Table 7:** Number of Salmonella outbreaks notified in cattle, Italy, 2011–2020 (updated to 13/01/2021).

**Year**	**North Italy**	**Central Italy**	**South Italy**	**Islands**	**Italy**
	**Number of outbreaks**	**Number of outbreaks**	**Number of outbreaks**	**Number of outbreaks**	**Number of outbreaks**
2011		1			1
2012	1	2		1	4
2013			1	1	2
2014				1	1
2015	3				3
2016	5			1	6
2017	6				6
2018	14			1	15
2019	26		1		27
2020	52			1	53
Total	107	3	2	6	118

In Italy there is not a national control program for *Salmonella* in cattle, thus data on the occurrence of Salmonellae in cows are collected in the framework of Directive 2003/99/EC. Data collected derive from research activities, official controls and clinical investigations, therefore, the general epidemiological situation may vary considerably over time.

The 2019 data uploaded by the IIZZSS network to the National Information System for Zoonosis (SINZOO) show a frequency of Salmonella detection of 0.3% (8/2,685) in official samples collected from cattle at the slaughterhouse, while a frequency of 10.1% (148/1,461) was recorded in samples (including organs, feces, milk and environmental samples) collected at farm level.

More than 80% of identified *Salmonella* strains belongs to three serotypes: *S*. Typhimurium (50.6%), *S*. Dublin (25.3%), and *S*. Typhimurium monophasic variant (9.6%).

The occurrence reported at slaughterhouse level (national data) is lower than what was observed in 2016 by Bonardi and colleagues (22), who detected *Salmonella* in 1.6% (95% CI: 0.4–5.6) of dairy cow carcasses randomly sampled at slaughterhouse.

Considering the increasing importance of salmonellosis in cattle and its potential impact not only on animals but also on public health, it is of pivotal importance to optimize the data collection system, as well as to standardize the methods used for epidemiological investigations in case of outbreaks.

### Q-Fever

Q-fever is a notifiable disease in Italy, but sanitary measures in infected herds must be applied only following the occurrence of human Q-fever cases (10). Despite the wide diffusion of *Coxiella burnetii* infection in the Italian cattle population, only five outbreaks of Q-fever have been officially notified in cattle in the last 10 years (2011–2020). A cross-sectional survey carried out to estimate the seroprevalence of *Coxiella burnetii* in extensively grazed cattle from Central Italy has detected a seroprevalence at the animal-level of 12.0, and 68.5% at animal- and herd-level, respectively (23). Large herd size, age and mixed breed scored as risk factors themselves for seropositivity in cattle (23). In North Italy a survey carried out using a PCR test on bulk milk samples reported a herd prevalence of infection of 43 and 60% if one or two checks are, respectively, applied (24).

Q-fever vaccines are available in Italy, but not frequently used.

### Neosporosis

Neosporosis is not a notifiable disease in Italy. There are no official data for the prevalence of this parasite, but *Neospora caninum* is widely present in the Italian cattle population. A serological survey carried out in Italy has detected a herd-level prevalence of 44.1%, and an animal-level prevalence about 11%. Neosporosis seroprevalence resulted higher in North Italy (25).

### Leptospirosis

Leptospirosis is a notifiable disease in Italy, and 45 outbreaks have been officially notified in cattle in the last 10 years (2011–2020), but the disease is probably underreported. In Italy about 10% of cattle with abortion are seropositive, and the prevalent serovar is *Leptospira Hardjo* (26). The disease is sporadically detected during diagnostic procedures on aborted fetuses and controlled with antibiotic treatments and/or autovaccines.

In case of a leptospirosis outbreak, all animal movements are officially blocked until the detection, treatment and full recovery of affected animals (10). Moreover, it is not allowed selling raw milk for direct human consumption produced by positive cows (10).

The National Reference Center for Leptospirosis has issued a draft guideline to standardize the approach to outbreak management.

### Epizootic Haemorragic Disease

Epizootic haemorrhagic disease has never been reported in Italy, however incursions of this disease in Italy are possible because EHD shares the same arthropod vectors (*Culicoides* spp.) with BT (27).

Vaccination is not allowed in Italy, because EHD is considered an exotic disease.

### Liver Fluke

Ruminant distomatosis (infestation caused by *Fasciola hepatica* or *Dicrocoelium dendriticum*) is a notifiable disease in Italy, however no outbreaks have been notified in the last 10 years (2011–2020). Liver fluke is present in the alpine area and in South Italy (4% prevalence in sheep flocks) where cattle are reared on pasture during the summer (28).

### Staphylococcus aureus

*Staphylococcus aureus* is the most important causative agent of subclinical mastitis in cattle, resulting in reduced milk production and quality. *S. aureus* infection is not a notifiable disease in Italy. There are no official data about the prevalence of this pathogen, but *S. aureus* is widely present in the Italian cattle population. A survey carried out in bulk tank milk in the region of Lombardy detected *S. aureus* in 47.2% of the tested dairy herds (29).

### Mycoplasma bovis

The *Mycoplasma bovis* infection is not a notifiable disease in Italy. There are no official data for the prevalence of *Mycoplasma bovis*, however we consider this disease endemic. A survey carried out on suckling dairy calves with respiratory disease detected a 31% prevalence of *M. bovis* infection (30); outbreaks of clinical mastitis have been reported as well (31).

### Mycoplasma mycoides

Contagious Bovine Pleuropneumonia (CBPP), caused by *Mycoplasma mycoides*, is a notifiable disease in Italy. Between October 1990 and October 1993, 94 CBPP outbreaks were notified in Italy (32), and the disease was eradicated thanks to a program, funded by the Government, based on active surveillance and a stamping out policy. Active surveillance activities had been in place until the end of 1995. Currently the country is considered free from *Mycoplasma mycoides* and is waiting for the status of officially free territory according to Reg. (EU) 2016/429. Vaccination against CBPP is forbidden.

### Surra (*Trypanosoma evansi*)

Surra sustained by *Trypanosoma evansi* has never been reported in Italy, and it is not a notifiable disease. However, other species belonging to the *Trypanosoma* genus were detected in the Italian cattle population. *Trypanosoma* (*Megatrypanum*) theileri was first reported in 1982 in healthy cattle of Central Italy (33), and more recently was incidentally detected in two sicilian cattle showing only a reduction in body weight (34). Infection with T. theileri in cattle normally results in a low parasitaemia probably limited by the host immune system and signs of disease are infrequent. However, parasite numbers in infected livestock can rapidly increase in immunocompromised, ill, or stressed animals (35).

### Aujeszky's Disease

In Italy a national control program for Aujeszky's disease (AD) is in place, based on compulsory vaccination of pigs. The disease is notifiable, and prevalence of infection in pigs has been decreasing in the last 5 years. AD also occurs in the wild boar population. No cases of clinical AD have been officially reported in cattle during the last 10 years (2011–2020), however sporadic cases, in particular in areas where pigs and cattle are grazing together, were recently reported (36).

### Trichophyton verrucosum

The disease is not notifiable in Italy. There are no official data about the prevalence of this dermatophytosis, however limited studies estimate a herd prevalence between 20 and 30% in North Italy (37), and much higher (60%) in Central Italy (38, 39).

An attenuated live vaccine is available in Italy, but its use is not extensive (38, 39).

### Bovine Coronavirus

The disease is not notifiable in Italy. The Bovine coronavirus was first detected in South Italy in 2008 (40), and recently it has been reported in the same area associated to a severe respiratory syndrome (41). Bovine coronavirus is probably endemic in the cattle population, but no official data are available about its prevalence.

### Bovine Respiratory Syncytial Virus

The disease is not notifiable in Italy. There are no official data for the prevalence of BRSV, however we consider this disease to be endemic since different BRSV strains are circulating in Italy (42, 43). Vaccination is widely used to control the disease, mostly in the beef sector.

### Bovine Digital Dermatitis

The disease is not notifiable in Italy. There are no official data for the prevalence of bovine digital dermatitis, however we consider this disease endemic, mainly in the dairy sector, and there are several veterinary practitioners expert in the treatment of this disease (44). Generally lameness is only considered an infectious problem of the single herd, but within the Classyfarm system described above, lameness is one of the animal-based measures of welfare assessment of cattle farms (7, 45).

## Discussion and Conclusions

The EU represents a huge market where live animals and animal products are exchanged between Member States without health barriers other than those defined by common rules, such as the Animal Health Law. Community regulation, however, covers only a part, considered as a priority, of the numerous infectious diseases affecting cattle and it may happen that within the 27 Member States there are other diseases with no or little EU regulation for which resources have been invested for their control for local interest. This is the case of the diseases selected in this report, for which there are CPs in at least two regions within the EU (2).

In Italy some of these diseases are covered by national or regional programs (Table 8). Currently the Italian Government has implemented national CPs only for EBL, BT, AD (in the pig sector) and JD. Because the Italian cattle population is not equally distributed throughout the country, it is not surprising that regional disease CPs (against IBR, BVD, *Streptococcus agalactiae*) have been implemented in regions of northern Italy, where 70% of the entire Italian cattle are raised. However, it should be noted that, generally, Italian farmers enroll in voluntary programs at a very low rate.

**Table 8:** Presence of Control programs in Italy for 24 cattle diseases with little no EU regulations.

**Disease**	**Listed in AHL**	**Notifiable in Italy**	**Control program in place (level)**	**Sector involved by the CP (dairy, beef, both)**
1. **Viral**				
Enzootic Bovine Leukemia	Yes	Yes	Yes (national)	Both
Bluetongue	Yes	Yes	Yes (national)	Both
Infectious Bovine Rhinotracheitis	Yes	No	Yes (national) Yes (regional)	Beef Both
Bovine Viral Diarrhea	Yes	No	Yes (regional)	Both
Epizootic Haemorragic Disease	Yes	Yes	No	
Aujeszky's Disease	Yes	Yes	Yes (national)	
Bovine coronavirus	No	No	No	
Bov. Respiratory Syncytial Virus	No	No	No	
2. **Bacterial**				
Bovine Paratuberculosis	Yes	Yes	Yes (national)	Both
*Streptococcus agalactiae*	No	Yes	Yes (regional)	Dairy
Anthrax	Yes	Yes		
Bov. genital Campylobacteriosis	Yes	No	Breeding bulls	Both
Salmonella	No	Yes	No	
Q-fever	Yes	Yes	No	
Leptospirosis	No	Yes	No	
*Staphylococcus aureus*	No	No	No	
*Mycoplasma bovis*	No	No	No	
*Mycoplasma mycoides*	Yes	Yes	No	
3. **Parasitic**				
Trichomonosis	Yes	Yes	Breeding bulls	Both
Neosporosis	No	No	No	
Liver fluke	No	Yes	No	
Surra (*Trypanosoma evansi*)	Yes	No	No	
4. **Mycotic**				
*Trichophyton verrucosum*	No	No	No	
5. **Other**				
Bovine digital dermatitis	No	No	No	

Although infectious diseases have a significant economic impact on cattle farms, until now both Italian farmers and Italian Health Authorities have given little importance to the control of non-zoonotic diseases. Recently, the market is increasingly requiring certifications on animal welfare and on the prudent use of antibiotics that cannot disregard the control of transmissible diseases. Following the implementation of a risk-based Official Control System and the increasing demand for antibiotic-free food, produced with respect for animal welfare, Italian farmers are probably going to invest more resources in farm biosafety and animal health in the near future. The recent developments and improvements of the national veterinary information systems will support this process by providing data on the health status of cattle farms.

## Data Availability Statement

The datasets presented in this article are not readily available because requests to access the diseases datasets should be authorized by the Italian Ministry of Health. Cattle Statistics are available at https://www.vetinfo.it/j6_statistiche/#/. Requests to access the datasets should be directed to https://www.vetinfo.it/.

## Author Contributions

MT, IP, and NP: conceptualization. MT, IP, SP, FF, CI, NA, DDS, AB, AS, and VC: writing. AS: figures editing. MT, AS, and IP: data collection and processing. LR and MI: supervision. All authors: review and editing.

## Conflict of Interest

The authors declare that the research was conducted in the absence of any commercial or financial relationships that could be construed as a potential conflict of interest.
