# Corrigendum: Overview of Control Programs for Twenty-Four Infectious Cattle Diseases in Italy

**DOI:** 10.3389/fvets.2021.818480

**Published:** 2021-12-07

**Authors:** Marco Tamba, Ivana Pallante, Stefano Petrini, Francesco Feliziani, Carmen Iscaro, Norma Arrigoni, Daria Di Sabatino, Antonio Barberio, Veronica Cibin, Annalisa Santi, Marco Ianniello, Luigi Ruocco, Nicola Pozzato

**Affiliations:** ^1^Istituto Zooprofilattico Sperimentale della Lombardia e dell'Emilia Romagna, Brescia, Italy; ^2^Istituto Zooprofilattico Sperimentale delle Venezie, Legnaro, Italy; ^3^Istituto Zooprofilattico Sperimentale dell'Umbria e delle Marche, Perugia, Italy; ^4^Istituto Zooprofilattico Sperimentale dell'Abruzzo e del Molise, Teramo, Italy; ^5^Ministry of Health, General Directorate of Animal Health and Veterinary Medicinal Products, Rome, Italy

**Keywords:** cattle, control programs, infectious diseases, Italy, SOUND-control project

In the title of the original article, there was an error. We used the phrase “EU non-regulated” for cattle diseases that are in fact listed in the New Animal Health Law (Regulation (EU) 2016/429) that went into force in 2021. The corrected title appears below.

**Title**

**Overview of Control Programs for Twenty-Four Infectious Cattle Diseases in Italy**

The authors apologize for this error and state that this does not change the scientific conclusions of the article in any way. The original article has been updated.

## Publisher's Note

All claims expressed in this article are solely those of the authors and do not necessarily represent those of their affiliated organizations, or those of the publisher, the editors and the reviewers. Any product that may be evaluated in this article, or claim that may be made by its manufacturer, is not guaranteed or endorsed by the publisher.

